# Clinical validity of fluorescence-based devices versus visual-tactile method in detection of secondary caries around resin composite restorations: diagnostic accuracy study

**DOI:** 10.1038/s41405-024-00284-7

**Published:** 2025-01-06

**Authors:** Aya Mohamed Adly, Shereen Hafez Ibrahim, Amira Farid El-Zoghbi

**Affiliations:** 1 Assistant lecturer at Conservative Dentistry, Faculty of Dentistry, Cairo University, Giza, Egypt; 2 Professor of Conservative Dentistry, Faculty of Dentistry, Cairo University, Giza, Egypt

**Keywords:** Dental lasers, Bonded restorations

## Abstract

**Objectives:**

To assess the validity of light-induced and laser-induced fluorescence devices compared to the visual-tactile method for detecting secondary caries around resin composite restorations.

**Materials and Methods:**

The study included 20 participants with 30 resin-composite restored teeth. Restorations’ margins were examined using three diagnostic methods: the visual-tactile method (FDI criteria), the light-induced fluorescence camera (VistaCam iX), and the laser-induced fluorescence device (DIAGNOdent pen), and the reference was visual inspection after removal of defective restorations. The validity of each method was evaluated. Inter-examiner reliability was calculated using Cohen’s kappa statistics. The level of significance was set at *P* = 0.05.

**Results:**

DIAGNOdent pen showed the highest sensitivity (100%) followed by VistaCam (98.82%) and the visual-tactile method (98.82%) at the enamel threshold. DIAGNOdent pen and VistaCam had lower specificity values than the visual-tactile method (81.69%, 76.06%, and 88.73% respectively). At the dentin threshold, DIAGNOdent pen yielded the highest sensitivity (89.36%), whereas VistaCam had the lowest (8.51%). The sensitivity of the visual-tactile method was low (57.45%) whereas all diagnostic methods had high specificity. There was perfect agreement in inter-examiner reliability for all assessment methods (Kappa 0.858–0.992).

**Conclusions:**

Both fluorescence-based devices and the visual-tactile method are reliable for detecting secondary caries around resin composite restorations. DIAGNOdent pen is accurate in enamel and dentin, while VistaCam and the visual-tactile method can detect secondary caries in enamel only.

**Clinical Relevance:**

Fluorescence-based devices could be used as a valuable aid to supplement or as a second opinion after the visual-tactile method.

**Trial registration:**

The study was listed on www.clinicaltrials.gov with registration number (NCT04426604) on 11/06/2020.

## Introduction

Secondary caries, defined as lesions at the margins of restorations, is considered the most common biological reason for failure in resin composite restorations, with an incidence ranging from 3.5% to 44% based on a recent systematic review [1]. Secondary caries primarily led to the replacement of about half of restorations, often resulting in overtreatment. Early detection of secondary carious lesions is considered a challenge among clinicians, as the change of color or marginal discrepancies around tooth-colored restorations are not all the time a predictor of secondary caries. These uncertainties could lead to incorrect decisions regarding restoration replacement. Moreover, detecting secondary caries sometimes occurs upon the removal of restoration, thereby accelerating the ‘cycle of re-restoration’ [2–4].

Researchers recognized that, up till now, there is no recommendation to any standard in the detection of secondary caries. Dentists use a variety of diagnostic methods, with great heterogeneity of subsequent treatment decisions. In addition, there is a trade-off between the specificity and sensitivity of diagnostic methods, resulting in over-detection or under-detection [5, 6].

Diagnostic methods and techniques to detect secondary caries around restorations were evaluated in terms of validity and reliability, in which a variety of both conventional and recent methods were discussed in literature [5, 6]. Conventionally, secondary carious lesions are assessed via visual-tactile examination, which is considered as the reference standard and the main method used for detecting secondary caries. Several detailed visual diagnostic systems have been proposed with the target of improving the accuracy and reliability of secondary caries detection around resin composite restorations [2–4]. Among the wide diversity of the visual-tactile assessment of the quality of restorations is the FDI criteria (Fédération Dentaire Internationale). A system presenting sixteen clinical criteria available, including esthetic, functional, and biological criteria, being one of those biological criteria, the “caries around restorations” [3, 7, 8]. However, visual-tactile assessment of caries did not fulfill the criteria for an ideal caries detection method due to their reliance on subjective interpretation and insensitivity to early caries detection. Therefore, the shortcomings of those conventional methods and the crucial need for supplementary methods have been acknowledged [9–11].

Among recent methods for caries detection were those methods based on fluorescence phenomena that have been applied to detect secondary lesions [5, 6]. Those devices that took the advantage of the fluorescence characteristics of tooth structure and caries process where carious tissue and healthy tissue emit fluorescence at different intensities when excited by light at specific wavelengths [10–12]. Moreover, when exposed to fluorescent-based light, most resin composite materials achieve a maximum fluorescence greater than that of tooth structure, making it easy to identify resin composite restorations. Therefore, fluorescence-based techniques could be powerful diagnostic tools for evaluating restoration margins [12, 13]. Among those fluorescence-based devices; DIAGNOdent Pen (Kavo, Biberach, Germany), which is a laser-induced fluorescence device that emits a diode laser having a wavelength of 655 nm. The other device is the VistaCam iX (Dürr Dental, Bietigheim-Bissingen, Germany), which is a light-induced intraoral camera that emits light having a wavelength of 400-nm [11, 14].

Upon reviewing the literature, it was found that the number of diagnostic accuracy studies assessing the validity of detecting secondary caries, especially studies on the application of fluorescence-based devices, is scarce. Most of those studies are in vitro studies, causing difficulty in generalizing findings, increased risk of bias, and concern about clinical applicability [5]. The literature includes only one systematic review assessing the clinical relevance of visual criteria in secondary caries detection [3]. Therefore, the dental literature lacks sufficient knowledge in this area, and evidence from clinical studies is needed to guide clinicians toward valid, reliable, and accurate diagnostic systems [6, 11, 14].

So, the current study was conducted to evaluate the clinical validity of both light-induced and laser-induced fluorescence devices in comparison to the visual-tactile assessment method in the detection of secondary caries around the margins of resin composite restorations. The null hypothesis was that there are no differences in the sensitivity, specificity, and accuracy of both fluorescence-based devices as compared to the visual-tactile assessment method when used to detect secondary caries around the margins of resin composite restorations.

## Materials and Methods

### Study setting

This clinical study was held in the Faculty of Dentistry, Cairo University, Egypt. All procedures performed in this study, involving human participants, were in accordance with the ethical standards laid down in the Faculty of Dentistry, Cairo University (Protocol registration number 04/06/2020) and in accordance with the Declaration of Helsinki and its later modifications. The protocol was registered at (www.clinicaltrials.gov), with the unique identification number NCT04426604 on 11/06/2020.

### Study design

**Fig. 1:**
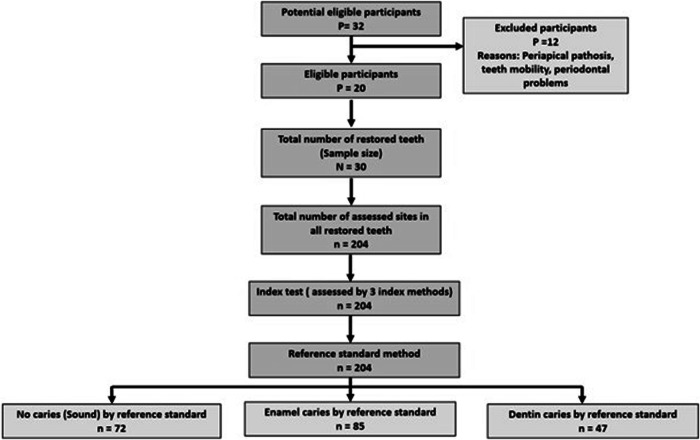
STARD Flow Diagram.

This study is a non-randomized diagnostic accuracy study with three-arms parallel design and was designed according to STARD (Standards for Reporting Diagnostic Accuracy Studies) flow diagram **(**Fig. 1**)**. The restorations were evaluated by three diagnostic methods: visual-tactile assessment method (FDI criteria), light-induced fluorescence intraoral camera (VistaCam iX), and laser-induced fluorescence device (DIAGNOdent pen 2190).

### Sample size calculation

A power analysis was designed to have adequate power to apply a statistical test of the research hypothesis that fluorescence-based devices would have similar diagnostic accuracy to the visual-tactile assessment method in secondary caries detection around margins of resin composite restorations. Based upon the results of Bamzahim et al. [15] which showed that the Area Under ROC (Receiver Operating Characteristic) curve (AUC) for DIAGNOdent was 0.78. The null hypothesis value was 0.5. Using Alpha (α) level of (5%) and Beta (β) level of (20%) i.e. power = 80%, the minimum estimated sample size was 30 teeth. Sample size calculation was performed using the software MedCalc Version 19.3.

### Randomization and Blinding

All participants’ resin composite restorations were evaluated by two examiners using all assessment methods, without any randomization. Each of the two examiners evaluated all investigation sites independently. Throughout the study, they were not allowed to exchange any data to avoid affecting the inter-examiner reproducibility [11, 16]. The examiners were not blinded to the index assessment methods. Prior to assessment using the fluorescence-based devices, the visual-tactile method was performed first to avoid any bias caused by knowledge of the findings from the other method. Afterward, the reference standard was implemented by the principal researcher.

### Calibration of examiners

The examiners in the current study were two expert clinicians having a minimum of 3 years of experience with each diagnostic method and should not have color weakness, or any grade of color blindness after assessment by the Ishihara test [12, 17]. The same examiners evaluated all investigation sites in all restored teeth.

Prior to the study’s initiation, calibration sessions were arranged for both examiners to achieve proper calibration at approximately 85% level to ensure reliable results [8, 14, 18, 19]. First, the examiners were calibrated on the FDI criteria using E-calib tool, where the cases were randomly selected from a database containing a large variety of high-quality professional photographs [8, 18]. The examiners observed those photographs of restorations that were representative of each score for each criterion. Then during the training sessions, twenty extracted teeth restored with resin composite restorations were collected to be assessed by the examiners using the visual-tactile method [4, 20]. Afterward, fluorescence images were taken of those restorations in the extracted teeth to calibrate the examiners on both fluorescence-based devices, a procedure that was done prior to the in vivo study [11, 21].

### Eligibility criteria for participants

Twenty participants were assigned where each of whom had at least one resin composite restoration (anterior or posterior), for a total of thirty restored teeth. The participants were recruited from an outpatient clinic of the Conservative Dentistry Department. Inclusion and exclusion criteria used for the enrollment of participants and restored teeth are presented in Table 1 [4, 22]. All the participants signed their written informed consent.

**Table 1:** Inclusion and exclusion criteria of participants and teeth.

Inclusion criteria	Exclusion criteria
**For Participants:**	**For Participants:**
• Adult participants aged from 18 to 60 years of age. • Participants had an acceptable oral hygiene level. • Participants had at least one resin composite restoration. • Participants accepted to participate in the trial.	• Participants with compromised medical history or active severe periodontal disease or with fixed orthodontic appliance.
**For restored teeth:**	**For restored teeth:**
• Resin composite restorations not involving proximal surfaces. • Teeth should be asymptomatic with no history of pain or symptoms of pulpal pathology. • Included restorations varied from having intact margins to defective or cavitated margins.	• Any restorations other than resin composite restorations. • Hopeless loose teeth with grade III mobility.

### Participants preparation

Before assessment of restorations, routine dental radiographs were taken to investigate the periapical area to exclude any periapical pathosis and to evaluate margins of the restoration at gingival margins as all these investigations would affect the final treatment decision. Then, scaling and polishing were done to the restored teeth using a rotating bristle brush at low speed without pumice or prophylactic paste, and then proper rinsing was done with a triple-way syringe for 10 seconds [14, 21, 23, 24]. Attention was given not to use the prophylactic paste on restored teeth because its remnants could significantly affect the accuracy of fluorescence-based methods [23, 25, 26]. Then teeth were isolated and dried using an air syringe for 5 seconds as a requisite for both the visual-tactile method and fluorescence-based devices [9].

### Participant assessment protocol

A total of 30 restored teeth from 20 participants were included in the study. Multiple sites at the restoration’s margins were assessed with a total of 204 sites was assessed in 30 included teeth. Those restorations’ margins ranged from intact margins, visual signs of demineralization, or cavitated margins [27].

### Visual-tactile assessment method using FDI criteria

**Fig. 2:**
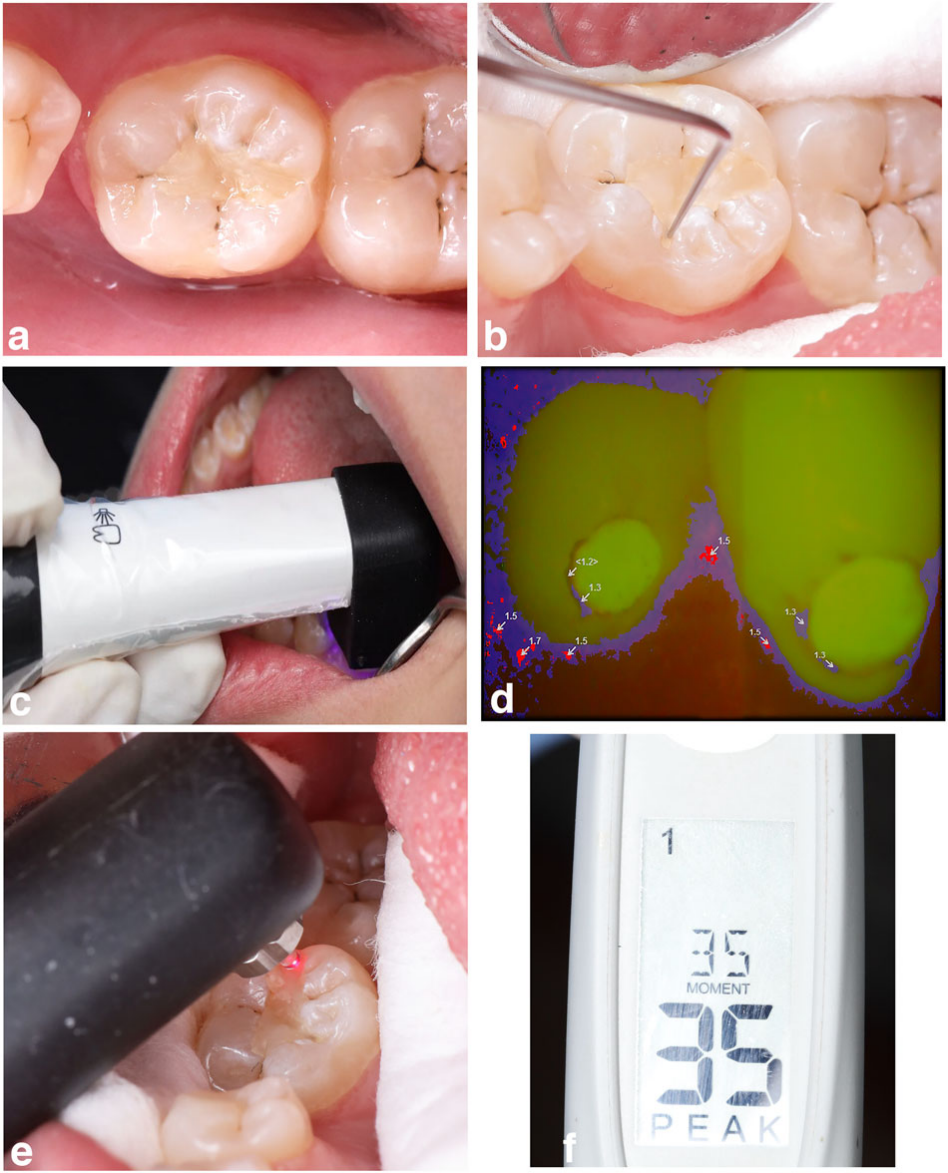
Index Assessment Methods. **a** Visual assessment according to FDI criteria under good illumination. **b** Tactile assessment to evaluate the margins of restorations using FDI probe. **c** The light-induced fluorescence camera VistaCam iX placed onto the tooth surface. **d** Analyzed images by the software of VistaCam iX. **e** The laser-induced fluorescence device (DIAGNOdent pen 2190) at resin composite restorations’ margins. **f** Readings obtained by the DIAGNOdent pen.

To standardize the conditions, all the diagnostic assessments were performed in the same dental units, with the same source of light, and in the morning [21]. Each participant was examined twice by the index assessment methods, once by each examiner (in separate dental units), and the examiners were unaware of each other’s results. The clinical examination of dental findings in each participant was done using a high-definition dental mirror (Hu-Friedy MfG. Co., LLC, Chicago, USA) and air-water spray. First, the examiners analyzed the restored teeth when wet and then when dry to detect any demineralization or carious lesions around restorations (biological criteria) as shown in Fig. 2a. Then FDI probe (Deppeler, Rolle, Switzerland) was used in this study according to FDI criteria as shown in Fig. 2b. The examiner outlined the extent of the restoration with all examined sites along margins on a sketch and rated a score for each criterion in a checklist (Table 2) [4, 7, 8].

**Table 2:** Scores of the index and the reference assessment methods.

Scores of the visual-tactile assessment method using FDI criteria	Scores of the light-induced fluorescence camera (VistaCam iX)	Scores of the laser-induced fluorescence device (DIAGNOdent pen 2190)	Scores of the reference Standard
**Score 1:** No caries/demineralization at the restoration margin detectable after air drying.	**Score 1:** 0.0–0.9 Green (sound enamel)	**Score 1:** 0–13 (Sound enamel)	**Score 1:** Sound
**Score 2:** First visible signs of a non-cavitated caries lesion at the restoration margin detectable after air drying	**Score 2:** 1.0-–1.4 Blue (early stages of enamel lesion)	**Score 2:** 14–20 (Enamel caries)	**Score 2:** Enamel caries
**Score 3:** Established, non-cavitated caries lesion or microcavity at the restoration margin detectable without air drying or larger areas of demineralization, but Only Preventive measures necessary (dentin not exposed).	**Score 3:** 1.5–1.9 Red (deep enamel lesion up to the dentin-enamel junction)	**Score 3:** 21–29 (Caries in the dentin-enamel junction)	
**Score 4:** Caries with cavitation, Localized dentin cavity (width > 250 μm, depth > 2 mm) at the restoration margin. Repair is possible (Localized and accessible can be repaired).	**Score 4:** 2.0–2.4 Orange (dentin caries dentin-enamel junction crossed)	**Score 4:**>29 (Dentin caries)	**Score 3:** Dentin caries
**Score 5:** Extensive dentin cavity at the restoration margin. Repair not possible/reasonable (not accessible for repair of restoration).	**Score 5:**>2.4 Yellow (deep dentin caries)		

### Fluorescence-based methods

For the light-induced fluorescence intraoral camera, VistaCam iX was placed in a specialized hygienic protective cover before use. The head of the camera was placed onto the tooth surface with the spacer connected onto it and enveloping the tooth surface. The intraoral daylight images were taken for the restorations using the daylight interchangeable head of the camera with the restored tooth under cotton roll isolation and after air drying for 5 seconds, then the fluorescence measurements were performed (Fig. 2c) [9, 11]. The software quantified the degree of fluorescence emitted by tooth tissues and analyzed the relationship between green and red fluorescent signals into values ranging from 0 to 3 as shown in Fig. 2d. Finally, the images with all values were saved, and the scores were categorized related to a range of numeric values, indicating the carious lesion’s severity (Table 2) [11, 24].

For the laser-induced fluorescence device DIAGNOdent pen 2190, the measurements were performed after calibration. Double calibration was done in which DIAGNOdent pen was first calibrated against the ceramic standard and then calibrated by being zeroed on sound enamel in each tested tooth. Then measurements were done with the teeth under cotton roll isolation after brief air-drying for 5 sec. The cylinder-shaped fissure probe of DIAGNOdent pen was used to carefully assess the tooth/restoration margins in which the probe is placed at the designed location perpendicular to the explored surface (Fig. 2e). Values would be displayed on the screen varying over a range of 0–99. Three measurements were performed consecutively for each site and the recorded measurement was corresponded to the highest value recorded in each site by two examiners (Fig. 2f). The results were interpreted according to the scale developed by the manufacturer (Table 2) [9, 11, 22, 28].

### Reference assessment method

For ethical reasons, the score of the visual-tactile method was considered solely as a reference standard if the case fulfilled all the following four conditions together: FDI score was less than 3, a VistaCam reading was less than 1.5 and a DIAGNOdent pen reading was less than 20 [29–31]. Then the score of the reference assessment method was recorded as follows: FDI: score 1, FDI: score 2, and FDI: score 3.

In cases other than those mentioned above, the reference assessment method was an assessment of the true extent of the secondary caries around the margins of restorations that was determined by the removal of this part of the restoration (if a small defect) or total removal of the restoration (if a large area or undermined) by the principal investigator.

Multiple sites at the restoration’s margins were assessed by all assessment methods, with a total of 204 sites in the 30 restored teeth. In the cases that could not be managed by refurbishing, then dealing with the defective restoration was through managing each defect alone, not the whole restoration at once [27, 32, 33].

**Fig. 3:**
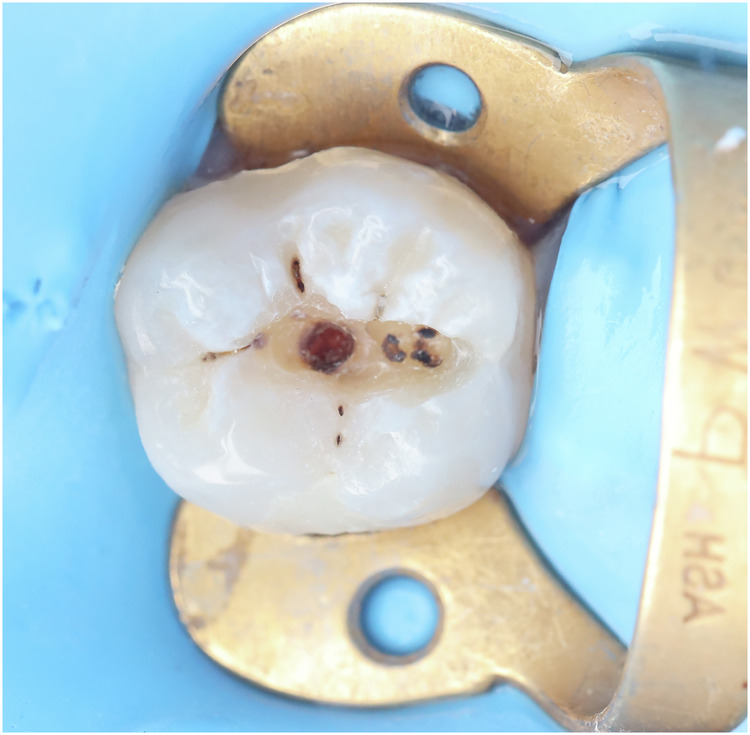
Reference standard assessment showing deep dentin caries after removal of the whole restoration.

For validation, the restorative material was carefully removed from the cavity using a tungsten carbide bur (Bur #245, Mesinger, Germany) at high-speed handpiece with copious water coolant on the expense of the restoration not the tooth. Great care should be taken to avoid contact of the bur with either of the cavity walls. The small remnants of restorative material in the cavity undercuts and the cavity walls were removed using a sharp excavator (Maillefer, Dentsply, Switzerland). The reference assessment method was inspection visually, and the final lesion depth value was measured, then the score of the reference assessment method was recorded [7, 34]. (Fig. 3, Table 2).

### Statistical analysis

Each examiner recorded the dental findings using all index assessment methods. To assess the validity of each diagnostic method in diagnosing secondary caries around margins of resin composite restorations in terms of sensitivity, specificity, accuracy, positive and negative predictive values (PPV and NPV, respectively) according to appropriate cut-off values. The diagnostic value of the diagnostic methods was determined by creating ROC curve, which is a graphic representation of the relationship between the sensitivity and 1-specificity. Then area under the curve (AUC) is a particular area present under the ROC curve, that directly assess the diagnostic power of the index assessment methods [9, 11, 35]. Youden’s index (J) was also calculated, which describes the actual effectiveness of the diagnostic method. The Youden index (J) is a function of sensitivity and specificity defined by J = maximum {sensitivity + specificity-1}. This index ranges between 0 and 1, with values close to 1 indicating that the method effectiveness is relatively large [11, 35].

Demographic data were represented in the form of frequencies and percentages (%) regarding gender of participants, included teeth and their distribution according to cavity class distribution [7, 36]. Pairwise comparisons were conducted to assess differences between the areas under the ROC curves and compared using z-statistics [9, 11, 35]. Qualitative data (scores) was presented as frequencies and percentages. The distribution of scores of each index assessment method was represented in comparison to the reference standard in the form of a cross-tabulation and analyzed using Chi-square test [7, 11]. The degree of inter-examiner reliability was calculated using Cohen’s kappa statistics [11, 37, 38].

The significance level was set at *P* = 0.05. All statistical analysis was performed with statistical software MedCalc® Version 14.8.1 for Windows and SPSS®IBM® Version 24 for Windows.

## Results

### Demographic data

Frequencies and percentages (%) for demographic data were presented in Table 3. The study included twenty participants, with a total of 30 restored teeth. Thirteen patients were females (65%) and seven patients were males (35%). Twelve anterior teeth (40%) and eighteen posterior teeth (60%) (four premolar teeth, fourteen molar teeth) were assessed. Seventeen restored teeth were class I resin composite restorations (56.6%), and thirteen were class V resin composite restorations (43.3%).

**Table 3:** Frequencies and Percentages (%) for demographic data.

		Frequency	Column %
**Gender**	Female	13	65%
	Male	7	35%
**Tooth**	Anterior	12	40.00%
	Posterior	18	60.00%
**Tooth distribution**	LL 3	1	3.33%
	LL 5	1	3.33%
	LL 6	4	13.33%
	LL 7	2	6.67%
	LL 8	1	3.33%
	LR 3	1	3.33%
	LR 4	1	3.33%
	LR 5	2	6.67%
	LR 6	5	16.67%
	LR 7	1	3.33%
	UL 1	2	6.67%
	UL 2	3	10.00%
	UL 3	1	3.33%
	UR 1	2	6.67%
	UR 2	2	6.67%
	UR 6	1	3.33%
**Cavity Class distribution**	Class I	17	56.67%
	Class V	13	43.33%

*LL* Lower Left, *LR* Lower Right, *UL* Upper Left, *UR* Upper Right.

### Validity

A total of 204 sites were assessed by all index methods, followed by the reference standard. The reference standard method revealed that 72 sites were healthy, 85 sites had enamel caries, and 47 sites had dentin caries. Diagnostic validity of index assessment methods was determined separately at various thresholds (related to reference standard): (1) No caries versus enamel caries (Enamel threshold), (2) No dentin caries versus dentin caries (Dentin threshold).

#### Validity at enamel threshold

Table 4 shows that sensitivity was the highest for DIAGNOdent, followed by VistaCam and the visual-tactile method using FDI criteria. It was found that the specificity values of the visual-tactile method were higher than those of both fluorescence-based devices.

**Table 4:** Area under the ROC curve, 95% CI, Sensitivity, Specificity, Accuracy, positive predictive value, negative predictive value and Youden’s index of the index assessment methods.

		Sensitivity (%)	Specificity (%)	AUC	95% CI	SE	PPV (%)	NPV (%)	Youden’s Index (J)	Accuracy (%)
**At Enamel Threshold**	Visual-tactile (FDI)	98.82	88.73	0.940	0.890–0.972	0.0192	91.306	98.432	0.8771	94.27
	VistaCam	98.82	76.06	0.874	0.812–0.922	0.0262	83.177	98.175	0.7521	88.54
	DIAGNOdent	100.00	81.69	0.898	0.839–0.940	0.0275	86.740	100	0.8194	91.72
**At Dentin Threshold**	Visual-tactile (FDI)	57.45	98.09	0.866	0.819–0.912	0.0238	90.474	87.951	0.555	88.73
	VistaCam	8.51	100.00	0.695	0.647–0.743	0.0247	100.00	77.584	0.335	79.31
	DIAGNOdent	89.36	96.82	0.951	0.912–0.976	0.0161	89.872	96.646	0.861	95.10

*AUC* Area Under ROC curve, *95% CI* 95% confidence interval, *SE* Standard Error, *PPV* Positive Predictive Values, *NPV* Negative Predictive Values.

Regarding ROC curve and Area under curve, it was found that the visual-tactile method had larger areas under ROC curve compared to both fluorescence-based devices with VistaCam having the smallest area under ROC curve.

The results indicated that the visual-tactile method outperformed fluorescence-based methods in terms of diagnostic accuracy. DIAGNOdent’s diagnostic accuracy was slightly better than VistaCam’s.

Regarding Youden’s Index (J), it was found that the visual-tactile method had greater J than those of both fluorescence-based devices, with VistaCam having the lowest value. Regarding predictive values, it was revealed that DIAGNOdent had the highest NPV, while the visual-tactile method had the highest PPV. VistaCam demonstrated the lowest PPV and NPV among methods.

The pairwise comparisons of Area under the ROC curve of different index assessment methods (Table 5) revealed that the difference in AUC between the visual-tactile method and VistaCam was significant (*P* = 0.0058). Whereas the visual-tactile method and DIAGNOdent showed an insignificant AUC difference (*P* = 0.1145). The difference in AUC between both VistaCam and DIAGNOdent devices was insignificant (*P* = 0.2008).

**Table 5:** Pairwise comparisons between Area under the ROC curve of index assessment methods.

**At Enamel threshold**	**Visual-tactile (FDI) and VistaCam**	
	Difference between areas	0.0654
	SE	0.0237
	95% CI	0.0190 to 0.112
	z-statistics	2.761
	**Significance level**	***P*** = 0.0058*
	**Visual-tactile (FDI) and DIAGNOdent**	
	Difference between areas	0.0422
	SE	0.0267
	95% CI	−0.0102 to 0.0945
	z-statistics	1.578
	**Significance level**	***P*** = **0.1145**
	**VistaCam and DIAGNOdent**	
	Difference between areas	0.0232
	SE	0.0181
	95% CI	−0.0123 to 0.0587
	z-statistics	1.279
	**Significance level**	***P*** = **0.2008**
**At Dentin threshold**	**Visual-tactile (FDI) and VistaCam**	
	Difference between areas	0.171
	SE	0.0262
	95% CI	0.119 to 0.222
	z-statistics	6.521
	**Significance level**	***P*** < 0.0001*
	**Visual-tactile (FDI) and DIAGNOdent**	
	Difference between areas	0.0851
	SE	0.0227
	95% CI	0.0406 to 0.130
	z-statistics	3.745
	**Significance level**	***P*** = 0.0002*
	**VistaCam and DIAGNOdent**	
	Difference between areas	0.256
	SE	0.0245
	95% CI	0.208 to 0.304
	z-statistics	10.456
	**Significance level**	***P*** < 0.0001*

^*^Significant, *SE* Standard Error, *95%* CI 95% confidence interval.

#### Validity at Dentin threshold

As presented in Table 4, the sensitivity was far higher for DIAGNOdent than all other index assessment methods. The visual-tactile method showed a low value of sensitivity. VistaCam had the lowest sensitivity. Regarding specificity, all index assessment methods revealed high specificity, with DIAGNOdent having the lowest specificity value.

Regarding ROC curve and Area under curve, it was found that VistaCam had the smallest Area under ROC curve compared with other methods, while DIAGNOdent had the largest Area under ROC curve.

It was shown that DIAGNOdent had the highest diagnostic accuracy among all assessment methods, while VistaCam had the lowest accuracy. The diagnostic accuracy of the visual-tactile method was in the middle range.

It was found that VistaCam’s Youden’s Index (J) was the lowest among index assessment methods, whereas DIAGNOdent had the highest value.

Regarding predictive values of all index assessment methods, it was revealed that VistaCam had the highest PPV, despite having the lowest NPV among methods. However, the visual-tactile method and DIAGNOdent had similar PPV values, and DIAGNOdent recorded the highest NPV values.

The pairwise comparisons of Area under the ROC curve of different index assessment methods (Table 5) revealed that the difference in AUC between the visual-tactile method and VistaCam was significant (*P* < 0.0001). Additionally, it was found a significant difference in AUC between the visual-tactile method and DIAGNOdent (*P* = 0.0002). The difference in AUC between VistaCam and DIAGNOdent was significant (*P* < 0.0001).

### Comparison between scores of index assessment methods and reference standard

Table 6 shows cross-tabulations for the distribution of scores of each index assessment method in comparison to reference standard that were analyzed using Chi-square test, where according to the results of the reference standard, 72 sites of restorations took score 1 (Sound), 85 sites took score 2 (Enamel caries) and 47 sites took score 3 (Dentin caries).

**Table 6:** Cross-tabulations for scores of the index assessment methods with the reference standard.

Frequency		FDI_Scores					
		1	2	3	4	5	Total Responses
**Reference Standard _Score**	**Sound**	64	6	2	0	0	72 (35.3%)
	**Enamel**	1	19	62	3	0	85 (41.7%)
	**Dentin**	0	6	14	26	1	47 (23.0%)
		65 (31.9%)	31 (15.2%)	78 (38.2%)	29 (14.2%)	1 (0.5%)	204
**Frequency**		**VistaCam_Scores**					
		**1**	**2**	**3**	**4**	**Total Responses**	
**Reference Standard _Score**	**Sound**	55	17	0	0	72 (35.3%)	
	**Enamel**	1	77	7	0	85 (41.7%)	
	**Dentin**	1	26	16	4	47 (23.0%)	
		57 (27.9%)	120 (58.8%)	23 (11.3%)	4 (2.0%)	204	
**Frequency**		**DIAGNOdent_Scores**					
		**1**	**2**	**3**	**4**	**Total Responses**	
**Reference Standard _Score**	**Sound**	59	11	0	2	72 (35.3%)	
	**Enamel**	0	54	28	3	85 (41.7%)	
	**Dentin**	0	1	4	42	47 (23.0%)	
		59 (28.9%)	66 (32.4%)	32 (15.7%)	47 (23.0%)	204	

According to the visual-tactile method using FDI criteria, it was found that 65 sites of restorations took score 1 (Sound), 109 sites took scores 2 and 3 (Enamel caries) and 30 sites took scores 4 and 5 (Dentin caries). The diagnosis, as determined by the measurements presented by VistaCam, revealed the following distribution: 57 sites of restorations took score 1 (Sound), 143 sites took scores 2 and 3 (Enamel caries) and only 4 sites took score 4 (Dentin caries). According to scores of DIAGNOdent, 59 sites of restorations took score 1 (Sound), 98 sites took scores 2 and 3 (Enamel caries) and 47 sites took score 4 (Dentin caries).

### Inter-examiner reliability of methods

Table 7 shows that Cohen’s Kappa value between the two examiners regarding the visual-tactile method using FDI criteria was 0.858 with 95%CI (0.811–0.905), which indicated substantial to perfect agreement between examiners. Regarding light-induced fluorescence intraoral camera (Vistacam iX), Cohen’s Kappa value between the two examiners was 0.992 with 95%CI (0.978–1.00), which indicated perfect agreement between examiners. Regarding laser-induced fluorescence Device (DIGANOdent pen), Cohen’s Kappa value between the two examiners was 0.992 with 95%CI (0.981–1.00), which indicated perfect agreement between examiners.

**Table 7:** Cohen’s Kappa value and 95% CI between readings of examiner 1 & examiner 2.

Method	Kappa Value	95% CI
Visual-tactile method (FDI criteria)	0.8585	0.8116 - 0.9054
Light-induced fluorescence intraoral camera (VistaCam iX)	0.9927	0.9786 - 1.0000
Laser-induced fluorescence device (DIAGNOdent pen)	0.9920	0.9811 - 1.0000

## Discussion

It was crucial to determine accuracy, sensitivity, and specificity to determine the most accurate assessment method with the most acceptable clinical performance for secondary caries detection around resin composite restorations. The null hypothesis was rejected, as there were differences in sensitivity, specificity and accuracy between both fluorescence-based devices and the visual-tactile method for detecting secondary carious lesions around resin composite restorations. The results of the current study revealed variability in the validity of secondary caries detection among methods depending on whether enamel or dentin thresholds. In the detection of enamel lesions, there were differences in specificity values between methods, with the visual-tactile method having the highest specificity values. In the detection of dentin lesions, there were large differences in sensitivity values between methods, with DIAGNOdent having the highest sensitivity values. It is worth mentioning that categorizing the FDI criteria into 3 categories, functional criteria (marginal adaptation), esthetic criteria (marginal staining), and biological criteria (recurrent caries), resulted in increased sensitivity and specificity values of secondary caries detection at the enamel threshold. Numerous studies validated this finding, showing that poor adaptation or marginal staining does not guarantee the development of caries around restorations [4, 7].

Despite the finding that in case of enamel lesions, the sensitivity values of the visual-tactile method were high, it was found that its sensitivity in the detection of dentin lesions was low. This is evident in the distribution of scores of FDI criteria compared to the reference standard. This could be the result of either undetected dentin carious lesions under the macroscopically intact surface (known as hidden caries) or an incorrect diagnosis of enamel caries, leading to the unchecked progression of dentinal lesions [23]. Similar findings were observed in other studies, where it was observed low sensitivity values for the visual-tactile criteria in detecting secondary dentin caries, resulting in more than half of teeth with secondary caries becoming misclassified as being sound via the visual-tactile method leading to false negative results [4, 7, 33, 39]. Contradictory results from another study indicated that visual criteria showed high sensitivity in detecting medium and large secondary carious lesions around restorations [22]. This study demonstrated that most medium and large secondary carious cavities might have gray discoloration displayed from demineralized dentin deep in the enamel of the cavity wall, a matter that made it easily detected through the visual-tactile method.

A Number of studies stated that fluorescence-based devices could reliably and accurately detect those lesions around resin composite restorations that might be useful to compensate the low sensitivity of visual-tactile method, a finding that was confirmed in the current study regarding DIAGNOdent, where it was found that the sensitivity of the device was the highest among all other assessment methods at dentin threshold [33, 37, 39, 40]. This finding was demonstrated in the distribution of scores of DIAGNOdent with the reference standard in the current study, a finding that was confirmed in a previous study [11]. Diniz et al. [22]. observed that sensitivity values of DIAGNOdent tended to increase in cases of advanced secondary carious lesions around resin composite restorations.

It is worthwhile mentioning that there are major difficulties in validating the diagnosis of secondary caries clinically when caries is present beneath the filling. Bamzahim et al. [41]. claimed that marginal integrity influences the sensitivity of fluorescence-based devices, in which caries might have gone undetected when the margins of the restoration are intact. This occurred in cases with deep caries at distant sites away from the restoration’s margin. They discussed how the access of the laser beam from DIAGNOdent may be obstructed by restorative material, making the detection of decay at a distance from the margins difficult. On the other hand, this wasn’t the condition in the current study, where almost all cases having deep carious lesions away from the margins were correctly classified as being carious by DIAGNOdent. Previous studies investigating secondary caries detection under resin composite restorations confirmed this finding, as DIAGNOdent demonstrated high sensitivity values [37, 42].

On the other hand, the current study showed that the light-induced fluorescence intraoral camera (VistaCam) displayed extremely low sensitivity values compared to DIAGNOdent and the visual-tactile method in detecting secondary carious lesions at the dentin threshold. The distribution of scores of VistaCam compared to the reference standard interpreted this extremely low sensitivity value at dentin threshold. Previous studies yielded similar findings, indicating that VistaCam could detect shallow lesions perpendicular to the capturing device, but it could not detect decay if the lesions were not perpendicular [11, 43]. But no previous studies investigated the sensitivity of VistaCam in detecting secondary carious lesions. On the other hand, VistaCam exhibited the highest specificity at the dentin threshold among the assessment devices, in which no cases had false positive results or were misdiagnosed as having dentin caries. These findings could be valuable in limiting the risk of overtreatment due to false positive results and thereby limiting the cycle of re-restorations.

It is worth noting that fluorescence-based devices’ specificity values vary depending on enamel or dentin thresholds. Although the specificity values of both VistaCam and DIAGNOdent were high at the dentin threshold, it was relatively low at the enamel threshold in both devices. The problem of low specificity of fluorescence-based devices that was confirmed in previous studies could be due to several factors, as marginal staining could be misdiagnosed as caries. Another cause could be the entrapment of saliva or plaque, organic deposits, or remnants of polishing pastes in marginal gaps, which could lead to a misdiagnosis of caries [15, 33, 39, 40, 44]. On the other hand, a number of studies demonstrated that the fluorescence-based devices had relatively high specificity values [22, 34, 42]. This contradiction could be due to combining the overall specificity of all lesions without categorizing enamel and dentin thresholds. In the current study, cases were categorized into enamel and dentin thresholds, knowing that the false-positive results occurred more in the enamel threshold (misdiagnosed as enamel caries).

Presenting those limitations such as relatively low specificity for enamel lesions and the necessity of absence of stains, plaque, and pastes during measurements, backed the view that these devices couldn’t be used in detecting or monitoring caries lesions of questionable activity [10]. In other words, they lack the ability of differentiation between active and inactive caries. A factor that is critical in clinical practice, as inactive (arrested) caries often do not require treatment. Distinguishing between active and inactive lesions is essential to avoid unnecessary interventions and the cycle of re-restoration triggered by false positives [10, 14].

Another important finding is that in the case of a marginal gap in a recent resin composite restoration without having incipient caries, the fluorescence-based devices might misdiagnose the plaque entrapped inside the gap as a carious lesion, leading to false positive results, while it could be easily detected by the visual-tactile method. It was important to clarify that the visual-tactile method showed good performance in accurate evaluation of those margins of resin composite restorations, especially those related to marginal staining and incipient color changes or marginal gaps in a recent restoration [22]. This was confirmed in the current study, where the specificity values of the visual-tactile method were higher than those of fluorescence-based methods. Therefore, the fluorescence-based devices could be considered a valuable tool for clinical detection of secondary dentin caries at margins around resin composite restorations along with the visual-tactile method.

A recommendation towards proper polishing, cleaning, and dryness of the restoration’s surface prior to the beginning of assessment of restorations to reduce the potential risk of diagnostic error [34, 42]. Contradictory opinions in literature were found, in which Brede et al. [45]. concluded that tooth polishing didn’t make a significant change in fluorescence values of the light-induced fluorescence camera. Similarly, Meller et al. [12]. claimed that the fluorescence-based devices were strong enough that neither plaque nor saliva had an impact on their performance. However, this was not the case in the clinical conditions where it was demonstrated that the performance of DIAGNOdent was relatively low under clinical conditions that could have been affected by oral environmental factors such as saliva and oral microflora, resulting in decreased accuracy and false results [26, 27, 34, 36].

Regarding the diagnostic accuracy in terms of area under the ROC curve (AUC), it was found that all index assessment methods showed relatively high AUC values at the enamel threshold with the visual-tactile method having the highest AUC value. This suggests their diagnostic usefulness in detection of secondary enamel carious lesions around resin composite restoration margins. On the other hand, the findings were different at the dentin threshold, in which DIAGNOdent showed the highest AUC value whereas the VistCam showed the lowest AUC value. Diniz et al. [22] found that DIAGNOdent presented higher AUC value than visual-tactile method, taking into consideration that the statistical analysis of AUC in that study was done without categorizing enamel and dentin thresholds.

When it came to pairwise comparisons between areas under the ROC curve of all assessment methods to analyze the significant differences between AUC areas. Starting with the enamel threshold, it was found a significant difference in AUC between VistaCam and the visual-tactile method. This finding aligned with the outcomes from a previous study [19]. On the other hand, the difference in AUC between the visual-tactile method and DIAGNOdent was insignificant. These findings came in agreement with those of previous studies [2, 34]. In addition, there is no significant difference in AUC between both fluorescence-based devices, which could be interpreted as having somehow similar performance regarding the detection of enamel lesions. Similar findings were obtained from previous studies comparing the performance of both DIAGNOdent and VistaCam in the detection of primary caries [11, 19, 43], but no studies in literature compared their performance in secondary caries detection. Regarding the dentin threshold, it was found that DIAGNOdent had the best diagnostic performance and validity in detection of secondary dentin lesions around resin composite restorations, as there was a significant difference in all pairwise comparisons between DIAGNOdent and the other assessment methods that ensured the poor performance of VistaCam and the visual-tactile method in detecting dentin lesions around resin composite restorations. This finding was previously confirmed on primary caries [43]. Comparing Youden’s index of the diagnostic methods revealed that the visual-tactile method showed the highest effectiveness in detection of enamel lesions, whereas DIAGNOdent showed the highest effectiveness in detection of dentin lesions. VistaCam revealed the lowest effectiveness in both thresholds.

Regarding inter-examiner reliability, the results of the current study showed that both examiners yielded perfect agreement in different assessment methods, with the fluorescence-based methods having slightly higher inter-examiner agreement. This high reliability justified that calibration and training sessions performed for all methods before the study were much more effective than personal experience, as no resulting differences were found between calibrated examiners with different experience. Similar results were obtained in other studies, in which the higher reliability of fluorescence-based methods was explained as being objective methods, being compared to visual- tactile assessment that relied on subjective aspects [2, 12, 28]. Accordingly, fluorescence-based methods demonstrate reduced variability in inter-examiner assessments, as it don’t rely on clinical experience level, making them advantageous in clinical research settings.

There was a debate about the importance of training and calibration sessions. Meller et al. [12] and Rodrigues et al. [39] claimed that the fluorescence-based devices didn’t need calibration sessions due to being highly reliable tools. On the other hand, Lenzi et al. [2] demonstrated that visual-tactile assessment was presenting lower reliability when not using standardized criteria or skipping training sessions. Similarly, Hamishaki et al. [28] and Ghoncheh et al. [40] asserted that the skills or experience of examiners did not influence the comparable inter-examiner reliability of both methods, provided they attended training sessions. Furthermore, the visual-tactile method using FDI criteria showed perfect inter-examiner agreement, a result that was consistent with a number of randomized clinical trials that discussed the reliability and sensitivity of FDI evaluation criteria to small variations in clinical outcomes [46–48]. Those findings were found to be a consequence of calibration and training sessions by the E-Calib tool to achieve high reliability between examiners [49].

The option for an appropriate method to detect secondary caries around restorations should be showing the best performance, both in terms of reliability and validity. When methods show near characteristics, the simplest and cheapest method should be preferred to maximize its use and spread it among dentists, increasing generalizability. Since DIAGNOdent demonstrated accurate measurements with high reliability for detecting both enamel and dentin secondary caries, it is a recommended means of secondary caries detection. However, most opinions would prefer their use in combination with conventional means as the visual-tactile methods, and care should be taken if using fluorescence-based measurements alone in making treatment decisions in clinical practice as there would be a risk of providing undertreatment or overtreatment. Therefore, it could be concluded that combining different systems could be the best approach for detecting secondary carious lesions around resin composite restorations. The null hypothesis was rejected, as there were differences in sensitivity, specificity and accuracy between both fluorescence-based devices and the visual-tactile method for detecting secondary carious lesions around resin composite restorations.

### Limitations of the study

Posterior resin composite restorations comprised a large part of the current study; therefore, it was challenging to create a dry field for secondary caries detection. Both fluorescence-based devices could have been affected by oral environmental factors, resulting in decreased accuracy and false results. Therefore, it was strongly recommended proper polishing, cleaning, and dryness of the restoration’s surface prior to the beginning of assessment to reduce the potential risk of diagnostic error [34, 37, 42]. Moreover, it is interesting to note that the use of fluorescence-based devices couldn’t give any information about lesion size, volume, depth or activity [33, 50]. As a result, it may be beneficial to combine different diagnostic systems for detecting secondary caries around resin composite restorations. In addition, it should be highlighted that the decision to repair or replace a restoration should also be based on other important factors, such as an individual’s caries risk, dietary habits, fluoride exposure, a reduction in carbohydrate intake frequency, or carious activity.

The current study investigated the performance of fluorescence-based methods in detecting secondary caries around Class I and Class V resin composite restorations, in which their practical utility in clinical practice might be limited. The relative consistency they provide might be more beneficial for research where standardization and repeatability are prioritized, but these advantages do not necessarily translate to routine clinical practice for Class I and V restorations, in which visual-tactile assessment is often adequate. Accordingly, further studies dealing with secondary caries detection in interproximal restorations should be implemented to show if another diagnostic methods might be recommended in conjunction with both assessment methods (visual-tactile method and fluorescence-based methods) to monitor their margins or any lesions around restorations.

## Conclusions

Within the limitations of the current study, the following conclusions could be drawn: Both fluorescence-based devices and the visual-tactile method are reliable for detecting secondary caries around resin composite restorations. DIAGNOdent pen is accurate in the detection of secondary caries in enamel and dentin. VistaCam can detect secondary caries in enamel only but has low validity in dentin that needs the use of adjunct diagnostic tools. The visual-tactile method using FDI criteria is accurate in the detection of secondary caries in enamel only.

### Clinical relevance

Each one of the diagnostic methods has its strengths and limitations; none of them exhibited perfect diagnostic performance to be recommended as a sole method for secondary caries detection around resin composite restorations. While fluorescence-based devices should not be used alone to make final treatment decisions, they can serve as valuable supplementary tools or second opinions in conjunction with visual-tactile methods.

## Supplementary information

STARD Checklist for Diagnostic Accuracy Studies

## Data Availability

The data that support the findings of this study are available from the corresponding author, upon reasonable request.
